# The transfer and decay of maternal antibody against *Shigella sonnei* in a longitudinal cohort of Vietnamese infants

**DOI:** 10.1016/j.vaccine.2015.12.047

**Published:** 2016-02-03

**Authors:** Corinne N. Thompson, Le Thi Phuong Tu, Katherine L. Anders, Nguyen Trong Hieu, Lu Lan Vi, Nguyen Van Vinh Chau, Vu Thuy Duong, Nguyen Ngoc Minh Chau, Tran Thi Hong Chau, Ha Thanh Tuyen, Tran Vu Thieu Nga, Pham Van Minh, Tran Do Hoang Nhu, Le Thi Quynh Nhi, Allan Saul, Laura B. Martin, Audino Podda, Christiane Gerke, Guy Thwaites, Cameron P. Simmons, Stephen Baker

**Affiliations:** a Oxford University Clinical Research Unit, Wellcome Trust Major Overseas Programme, Ho Chi Minh City, Viet Nam; b Centre for Tropical Medicine, Nuffield Department of Clinical Medicine, Oxford University, Oxford, UK; c London School of Hygiene and Tropical Medicine, London, UK; d School of Biological Sciences, Monash University, Victoria, Australia; e Hung Vuong Hospital, Ho Chi Minh City, Viet Nam; f The Hospital for Tropical Diseases, Ho Chi Minh City, Viet Nam; g Novartis Vaccines Institute for Global Health^2^, A GSK Company, Siena, Italy; h Department of Microbiology and Immunology, University of Melbourne, Australia

**Keywords:** *Shigella*, Maternal antibody, Placental transfer, Seroconversion

## Abstract

**Background:**

*Shigella sonnei* is an emergent and major diarrheal pathogen for which there is currently no vaccine. We aimed to quantify duration of maternal antibody against *S. sonnei* and investigate transplacental IgG transfer in a birth cohort in southern Vietnam.

**Methods and results:**

Over 500-paired maternal/infant plasma samples were evaluated for presence of anti-*S. sonnei*-O IgG and IgM. Longitudinal plasma samples allowed for the estimation of the median half-life of maternal anti-*S. sonnei*-O IgG, which was 43 days (95% confidence interval: 41–45 days). Additionally, half of infants lacked a detectable titer by 19 weeks of age. Lower cord titers were associated with greater increases in *S. sonnei* IgG over the first year of life, and the incidence of *S. sonnei* seroconversion was estimated to be 4/100 infant years. Maternal IgG titer, the ratio of antibody transfer, the season of birth and gestational age were significantly associated with cord titer.

**Conclusions:**

Maternal anti-*S. sonnei*-O IgG is efficiently transferred across the placenta and anti-*S. sonnei*-O maternal IgG declines rapidly after birth and is undetectable after 5 months in the majority of children. Preterm neonates and children born to mothers with low IgG titers have lower cord titers and therefore may be at greater risk of seroconversion in infancy.

## Highlights

• *Shigella sonnei* is an emergent and highly drug resistant diarrheal pathogen.
• The half-life of maternal *S. sonnei* IgG in infants is 43 days.
• Maternal titer, antibody transfer ratio and gestational age influence birth titer.
• Incidence of seroconversion in infants in southern Vietnam is 4/100 infant years.
• Children should be vaccinated after 5 months of age if a candidate is licensed.

## 1 Introduction

The bacterial genus *Shigella* is a major contributor to the global burden of diarrheal disease. This genus of enteric pathogens is typically associated with disease in children under 5 years of age in industrializing regions [1], and is estimated to be responsible for 100,000 deaths annually [2]. *Shigella* infections are characteristically associated with dysentery (blood and mucus in the stool) and can be severe in young children [3], [4]. Of the four *Shigella* species, *Shigella flexneri* and *Shigella sonnei* predominate worldwide [1]. *S. flexneri* is traditionally associated with disease in industrializing countries, whereas *S. sonnei* is more commonly isolated in industrialized regions. However, this distribution is changing. *S. sonnei* is globally emergent and replacing *S. flexneri* as the most common cause of bacterial dysentery [5], [6]. This trend may be being exacerbated by resistance to common antimicrobials, with several recent reports of *S. sonnei* exhibiting resistance against fluoroquinolones and 3rd generation cephalosporins in the USA, Vietnam and elsewhere [7], [8], [9]. Improved sanitation and antimicrobial treatment remain the only current tools for prevention and control as there are no licensed *Shigella* vaccines [10].

Neonates and infants are typically at increased risk from infectious agents such as *Shigella* due to immaturity of the immune system [11]. While neonates have some capacity for cell-mediated immunity [12], humoral immunity is very limited in early life [13]. Antibody responses in neonates are shorter, delayed in onset and of lower affinity than those observed in healthy adults [14]. The transfer of maternal IgG antibody to the fetus during pregnancy confers short-term passive immunity and represents a primary mechanism for protection against infectious diseases at birth [11]. Transport of maternal antibody across the placenta to fetal capillaries is mediated by the neonatal Fc receptor (FcRn) [15], [16], [17] and can be affected by factors such as gestational age, maternal IgG concentration and infection [18], [19], [20], [21].

Maternally transferred IgG against *S. sonnei* in infancy has not been substantially investigated. Work conducted in Israel in the mid-1990s found that the concentration of anti-*S. sonnei* lipopolysaccharide (LPS) IgG present in umbilical cord plasma positively correlated with the concentration in maternal plasma [22]. IgG against LPS, specifically the O-antigen component, is the best described *S. sonnei* immune marker as it is the major bacterial surface antigen exposed to the immune system during infection. Although anti-*S. sonnei*-O IgG is not a definitive correlate of protective immunity [23], it is an indicator of some degree of acquired immunity; lack of *Shigella* serotype specific antibody is associated with an increased risk of symptomatic disease [24], [25]. Furthermore, titers of anti-*S. sonnei*-O IgG rise significantly after symptomatic infection [22], [26], [27], with titers doubling 10 weeks post-infection [26], [28]. Previous work from Vietnam in the late 1980s showed that anti-*S. sonnei*-LPS and anti-*S. flexneri*-LPS IgG rise dramatically from birth, peak at 3–4 years of age and then permanently plateau [29].

An understanding of the nature and duration of maternal antibody protection in infancy is important for determination of an appropriate vaccination schedule when *Shigella* vaccines eventually become available. Additionally, although IgG titers against *S. flexneri* and *Shigella dysenteriae* type I in Vietnam were found to be high in children and adults in the early 1990s [27], [29], exposure to *Shigella* has not been measured in a contemporary Vietnamese population. As *S. sonnei* is now the predominant *Shigella* species in Vietnam [30], we hypothesized there would be substantial evidence of population exposure and *S. sonnei* maternal antibody transfer in this rapidly industrializing country. Therefore, we aimed to quantify maternal anti-*S. sonnei*-O antibody decay using the largest sample size to date, with over 500 paired mother and infant plasma samples. We also investigated transplacental IgG transfer and determined the incidence of *S. sonnei* seroconversion in infancy in southern, urban Vietnam.

## 2 Methods

### 2.1 Ethical approval

Written informed consent was required from all enrolled families. Ethical approval was granted from Hung Vuong Hospital, Oxford Tropical Research Committee as well as the London School of Hygiene & Tropical Medicine for the main cohort study. Ethical approval was also granted from the Hospital for Tropical Diseases in HCMC and OxTREC for the studies collecting acute and convalescent plasma samples from culture-positive *Shigella* and *Salmonella* cases for ELISA validation.

### 2.2 Study population

The birth cohort population and methodology has been described previously in detail [31]. Briefly, mothers delivering at Hung Vuong obstetric hospital in Ho Chi Minh City (HCMC) were invited to enroll during either an antenatal visit in the final month of pregnancy or at the time of hospital admission for delivery. Children born between January and December 2013 in HCMC were included in the analysis presented here. Pregnant women were eligible if they lived in district 8 of HCMC (a previously identified endemic hotspot for *Shigella* [30]), were aged 16 years or older and were HIV seronegative at the time of birth. Mothers answered a baseline questionnaire and blood (umbilical cord and venous) samples were collected in EDTA tubes. After delivery, infants were recalled regularly for routine follow up visits. A 1 ml EDTA blood sample was collected at the 4, 9 and 12 month visits. All blood samples were separated into cells and plasma and stored at −20 °C until required.

### 2.3 *S. sonnei* anti-O antigen ELISA

Antibody (IgG and IgM) against *S. sonnei* O-antigen were measured using an enzyme-linked immunosorbent assay (ELISA) in maternal, umbilical and longitudinally collected infant plasma samples. Purified *S. sonnei* O-antigen was extracted as previously described [32]. For the ELISA assays, 96-well microtiter plates (Maxisorb; NUNC) were coated overnight with 0.5 g/ml *S. sonnei* O-antigen in PBS pH 7.0 at 4 °C, plates were then washed and blocked in PBS containing 5% skimmed milk powder for 2 h. After washing, 100 μl of each plasma sample (diluted at 1:200 in PBS containing 1% skimmed milk) were added and plates were incubated for 2 h at room temperature. IgG and IgM against *S. sonnei* O-antigen were detected by incubation with alkaline phosphatase directly conjugated anti-human IgG/IgM for 1 h. Plates were developed by p-nitrophenyl-phosphate solution (Sigma) and were read at absorbance 405 nm and 490 nm by an ELISA platereader (Microplate reader, Biorad). Each plate contained a 2-fold serially diluted pool of anti-*S. sonnei*-O antigen human plasma (primary concentration 1:200). A standard curve was generated from the corresponding optical density (OD) and ELISA units using a 4-parameter logistic regression fit. One ELISA unit (EU) was defined as the reciprocal dilution of the standard plasma that gave an absorbance value equal to 1 in this assay. The ELISAs were done in duplicate. Antibody (IgG and IgM) units in the cohort members’ plasma were calculated relative to this standard each time the assay was performed. Acute and convalescent plasma samples for the ELISA validation were derived from pediatric culture-positive *S. sonnei* and *Salmonella* dysentery cases presenting to either the Hospital for Tropical Disease in HCMC as part of another ongoing study.

### 2.4 Statistical analyses

Geometric mean titers (GMT) were calculated to summarize anti-*S. sonnei*-O IgG in maternal and cord plasma. Paired *t*-tests were used to compare log_10_ titers between paired maternal/cord samples. Analysis of variance (ANOVA) with Bonferroni correction for multiple comparisons was used to compare maternal and cord log-transformed antibody titers within categorical groups. The ratio of maternal transfer was compared across groups using the Kruskal–Wallis (KW) test with Dunn's test for multiple comparisons [33]. Linear mixed effects modeling was used to assess the trajectory of infant log_10_ titers from birth to 20 weeks to account for within-participant association over time. The half-life of IgG titer was calculated as the time at which the predicted IgG titer would decrease by 50% from the cord blood titer. The population half-life was derived using the formula:

$$\frac{-\log_{10}(2)}{b_{1}}$$

with *b*_1_ equal to the slope of the fixed effect. The 95% confidence interval (CI) for the population level half-life was derived from the CI of the slope of the fixed effect. Children with a 4-fold rise between serial titers or those who were aged <6 months without a decrease in IgG titer were censored after the time point prior to the increase or no decrease, respectively [34].

A Kaplan Meier survival curve was generated to investigate the time taken for titers to fall below a detectable threshold of 10.3 EU. This threshold value was determined by calculating the mean titer value of the observation preceding a 4-fold rise in titer in infants that had a 4-fold rise with a gap between pre and post-seroconversion samples no greater than 24 weeks (*n* = 15). None of the cord plasma titers and less than 1% (2/502) of the maternal plasma samples had titers that fell below 10.3 EU. For the Kaplan Meier estimation, infants were censored either when they (1) dropped below 10.3 EU (2) had any rise in IgG titer or (3) were lost to follow up. Finally, linear regression was used to evaluate the effect of covariates on anti-*S. sonnei*-O IgG cord titer as well as the relationship between log_10_ cord titer and log_10_ increase in titer between serial follow up visits. All analyses were performed in STATA v13 (TX, USA) with the exception of the mixed effects modeling which was performed in R (version 3.0.2) using the lme4 package [35]. Plots were made in R using the ggplot package v1.0.1 [36].

## 3 Results

### 3.1 ELISA validation

We firstly validated the anti*-S. sonnei*-O ELISA in a population of Vietnamese children hospitalized with dysentery with acute and convalescent plasma samples. All tested (7/7; 100%) stool culture-positive *S. sonnei* cases presenting to hospital had >4 fold rise (median: 104-fold; range: 22–410) in IgG titer regardless of the number of days between the acute and convalescent samples (median: 116 days; range: 13–202). The IgM titers against *S. sonnei* O-antigen of the seven responding children also increased dramatically (median: 9-fold, range:3–64). Twenty culture positive *Salmonella* cases from the same study did not generate an *S. sonnei* O-antigen IgG response (median fold titer increase: 1.1, range: 0–2.0), with limited IgM response as well (median: 1.4-fold, range: 0–58) (data not shown).

### 3.2 Cohort baseline characteristics

**Fig. 1:**
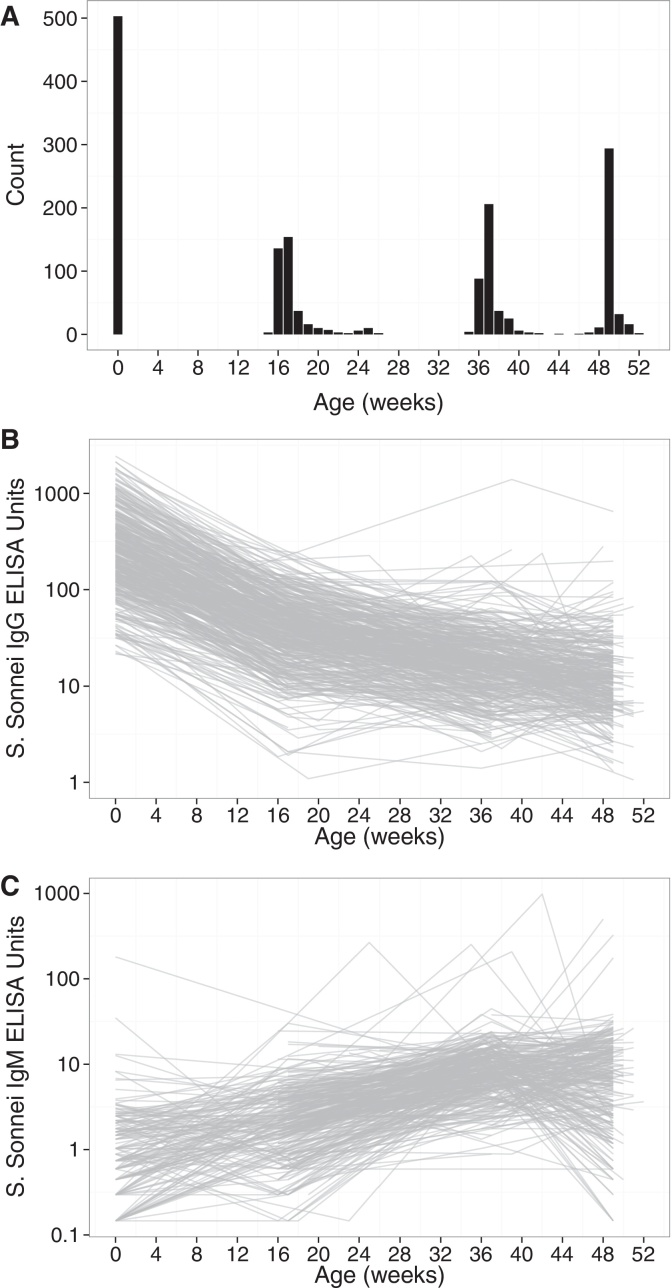
Anti-*S. sonnei*-O antibody levels in the first year of life in a cohort of 503 Vietnamese children. (A) Count of the number of assayed infant plasma samples at different ages in the first year after birth. Anti-*S. sonnei*-O IgG (B) and IgM (C) titers shown over time for each individual in the cohort on a log_10_ scale.

Of the 503 infants enrolled into the birth cohort in 2013, 52% (260/503) were male, 4% (21/503) were born preterm (<37 weeks of gestation) and 5% (23/503) were of low birth weight (<2.5 kg) as shown in Table 1. The median maternal age was 28 years (interquartile range (IQR): 25–31), with just under half of all mothers (244/503; 49%) reporting at least a higher secondary education. The median maternal gravidity was 2 (IQR: 1–3) and the mean duration of infant follow up was 337 days (range: 1–399 days). A total of 58% (292/503) infants enrolled returned for all three follow up appointments where a blood sample was collected (Fig. 1A). A further 78% (393/503) returned for at least two blood-draw appointments, and 86% (432/503) for at least one follow up blood-draw appointment. There were no major demographic or socioeconomic differences between the families of infants who did not return for all four follow up visits (211/503; 42%) and those that did return for all four visits (292/503; 58%) (Table 2).

### 3.3 The decay of maternal anti-*S. sonnei*-O IgG and incidence of seroconversion

**Fig. 2:**
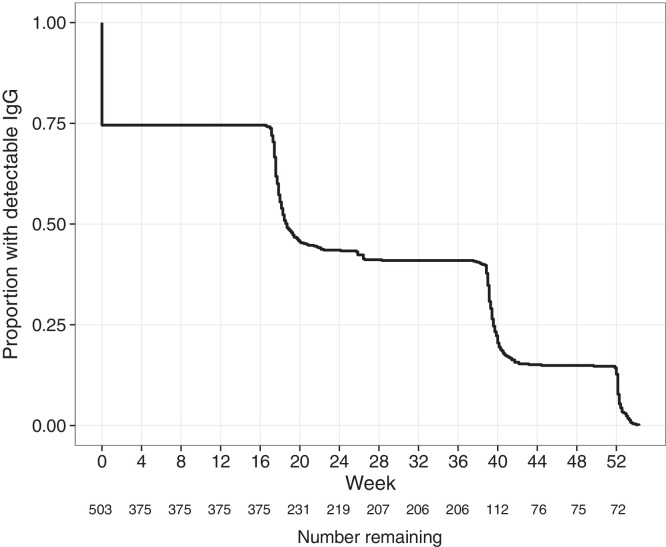
Kaplan Meier curve showing the proportion of infants with detectable anti-*S. sonnei*-O IgG in the first year after birth. The proportion of infants with detectable anti-*S. sonnei*-O IgG censored by (1) when their titer dropped below 10.3 EU (see methods), (2) had any detectable increase in IgG titer or, (3) lost to follow up. The number of infants with detectable antibody at each time point are shown below the *x*-axis.

The anti*-S. sonnei*-O IgG and IgM titers in infants over the first 12 months of life are shown in Figs. 1B and C, respectively. Using samples collected within 20 weeks of birth, we estimated the median half-life of anti-*S. sonnei*-O IgG to be 43.2 days (95%CI: 41.9–44.5 days). As shown in Fig. 2, by 18.7 weeks (95%CI: 18.1–20.1 weeks) 50% of infants had undetectable levels of anti-*S. sonnei*-O IgG. A total of 16 children had a >4-fold rise in anti-*S. sonnei*-O IgG titer in the first year after birth (3.2%), the majority of which occurred between 4 and 9 months (8/16, 50%), or 9 and 12 months (6/16, 38%) after birth (Fig. 2). Critically, a higher fold-rise in anti-*S. sonnei*-O IgG over the first 12 months of life was associated with a lower cord titer (*p* < 0.001; linear regression). There were 463.5 infant years of follow up in this cohort, leading to a seroconversion rate (defined by >4-fold rise in titer) of 3.5/100 years of follow up in the first 12 months of life. Two children did not have a detectable decrease in IgG titer between birth and 20 weeks of life (0.4%). Furthermore, 49/503 infants (10%) had a 2-fold rise in anti-*S. sonnei*-O IgG over the course of the first 12 months of life, the majority of which (25/49, 51%) occurred between 9 and 12 months of age. Out of the 503 infants enrolled in the cohort, 162 (32%) had a rise (any) in titer over the first year of life, which were more commonly detected between 9 and 12 months of age (84/162; 52%).

### 3.4 Maternal antibody transfer

The geometric mean titers of anti*-S. sonnei*-O IgG in cord plasma and maternal plasma were 234.1 EU (range: 21.6–3687.6 EU) and 167.4 EU (range: 3.75–2553 EU), respectively (Table 2). The median ratio of cord:maternal plasma anti*-S. sonnei*-O IgG was 1.32 (range: 0.3–12.4) (Table 2). Anti*-S. sonnei*-O IgG titers in cord plasma were consistently and significantly higher than those in maternal plasma (Table 2), with the exception of babies born preterm (*p* = 0.71, paired *t*-test of log_10_ titers). The ratio of maternal transfer in preterm babies (median: 1.13) was significantly lower than in babies born 37–40 weeks (median: 1.35) (*p* = 0.02; KW). Furthermore, the transplacental transfer ratio in first-pregnancy mothers (median: 1.38) was moderately higher than in mothers that had had previous pregnancies (median: 1.30, *p* = 0.066; KW). The maternal transfer ratio was also slightly greater in younger mothers (<28 years) than in mothers ≥28 years of age (median: 1.36 versus 1.29, respectively; *p* = 0.065; KW). Finally, the transplacental transfer ratio was significantly higher in infants born in January–March (median: 1.70) and in April–June (median: 1.90) compared to those born in July–September (median: 1.14) and October–December (median: 1.12) (Table 3).

### 3.5 Factors influencing anti-*S. sonnei*-O cord blood antibody titers

**Fig. 3:**
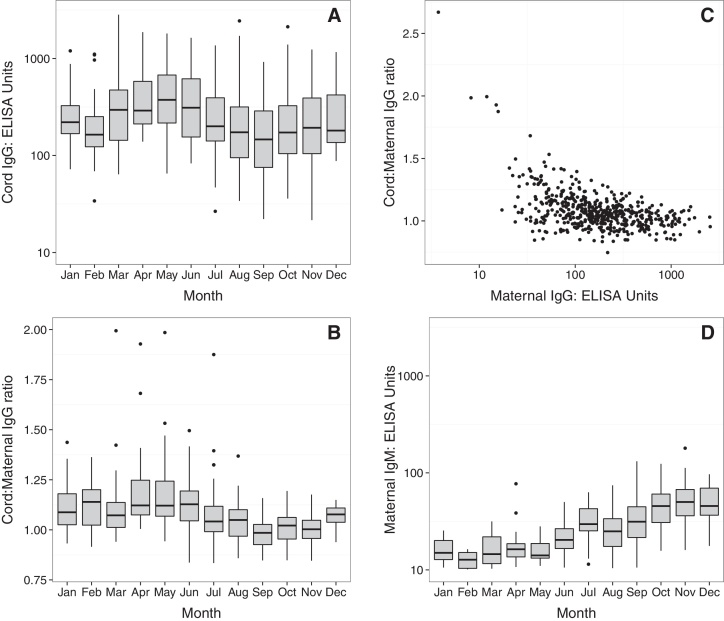
Temporal anti-*S. sonnei-*O antibody cord titers and transplacental transfer dynamics. (A) Anti-*S. sonnei*-O IgG cord plasma titers shown by month of birth on a log_10_ scale. (B) The ratio of cord:matneral anti-*S. sonnei*-O IgG titer by month of birth. (C) Scatterplot showing the relationship between maternal anti-*S. sonnei*-O IgG titers and the ratio of cord:maternal plasma transfer. (D) Maternal anti-*S. sonnei*-O IgM titers shown by month of birth on a log_10_ scale.

Anti-*S. sonnei*-O cord IgG titer was associated with several covariates in a univariate analysis (Table 3). However, after controlling for the effects of confounding in an adjusted analysis the only covariates that remained significantly associated with anti-*S. sonnei*-O cord IgG titer were ratio of transplacental IgG transer, maternal IgG titer and the season of birth. As shown in Fig. 3A, anti-*S. sonnei*-O cord IgG titers in babies born in the first half of the year were higher than those born in the second half of the year. The ratio of anti-*S. sonnei*-O IgG maternal transfer was also elevated in the first half of the year compared to the later months (Fig. 3B). Significantly, the ratio of transplacental transfer was higher in mothers with low IgG at the time of birth (Fig. 3C) (*p* < 0.001; linear regression of log_10_ maternal titers) (Table 4).

On additional analysis we found that maternal IgM increased (Fig. 3D) from May to July, plateauing in the later months of the year, suggesting that some mothers were likely exposed to *S. sonnei* at the time of birth during April-June. Maternal IgG levels did not significantly change throughout the year, although the titers were generally high, suggesting previous and potentially sustained exposure. The combination of low existing maternal IgG in some mothers during April–June and the increased the ratio of transplacental transfer during this period lead to an overall elevated anti-*S. sonnei*-O IgG in babies born during this period, which may be during a period of increased seasonal *S. sonnei* transmission in HCMC.

## 4 Discussion

*S. sonnei* is an emergent and increasingly antimicrobial resistant diarrheal pathogen. As such *S. sonnei* is a growing challenge in Vietnam and other similarly industrializing countries [5], [6], [37], [38], [39], [40], [41], [42]. The aims of this study were: (1) to quantify the duration of maternal IgG in infants, (2) to measure incidence of *S. sonnei* seroconversion in the first year of life and (3) to examine transplacental IgG transfer during pregnancy. As *S. sonnei* vaccines are in development [23], understanding the potential of maternal immunity in infants will be critical for evaluating future vaccine efficacy and identifying the infant groups that are most at risk of *S. sonnei* seroconversion [4].

The estimated half-life of maternal anti-*S. sonnei*-O IgG (43 days, 95%CI: 42–45 days) is similar to that of *Haemophilus influenza*e (33 days), pertussis (36–40 days) and *S. pneuomoniae* (35 days) [43], [44], [45]. However, as the sampling was infrequent in the early weeks after birth these data should be interpreted with caution. Nevertheless, it is apparent that maternal antibody wanes rapidly and by 5 months of age the majority of infants had no circulating maternal antibody and are likely at increased risk of infection. Correspondingly, evidence of *S. sonnei* exposure in infants in our cohort suggests an incidence of seroconversion of approximately 4/100 infant years of follow up in HCMC. Yet given the known lack of general humoral immune responses against polysaccharides during infancy [13], in addition to loss to follow up, this seroconversion incidence is likely an underestimate.

We found that lower cord titers were associated with higher fold-increases in anti-*S. sonnei*-O IgG titer in the first year of life in our cohort, suggesting that neonates born with lower cord titers are at increased risk of seroconversion during infancy. The most important influences on anti-*S. sonnei*-O cord titer were maternal IgG titer and the ratio of transplacental transfer, which were inversely correlated. Such a relationship is due in part to saturation of the Fc receptor, as IgG that is not bound is digested by lysosomal enzymes inside the syncytiotrophoblast [11], [46]. The negative relationship between maternal IgG concentration and transplacental transfer ratio has been suggested to demonstrate the existence of a mechanism to protect the newborn through strengthening the transfer of antibody when maternal levels are not optimally protective [47], [48]. Furthermore, it has been found that a higher total maternal IgG concentration may lead to reduced transfer efficiency of both total and specific IgG [21], with some suggestion of receptor competition among antigen-specific IgG for the limited number of placental Fc receptors available [20].

Neonates tended to have elevated anti-*S. sonnei*-O IgG titers compared to mothers in our cohort. Such a phenomenon has been reported for a variety of pathogens including *Klebsiella pneumoniae*, *Escherichia coli* and *Pseudomonas aeruginosa* [18], [47], [49]. However, neonates born preterm in this cohort did not have an increased anti-*S. sonnei*-O titer relative to their mothers. As the majority of IgG is acquired by the fetus during the last 4 weeks of pregnancy [50], it follows that preterm neonates would lack maternal immunity and are potentially at increased risk for infections in the first few months of life. Furthermore, we found that children born to mothers with lower IgG titers had lower cord titers themselves and are at increased risk of exposure.

Interestingly, we noted a seasonal pattern to both cord plasma titers as well as the ratio of transplacental transfer in our cohort. Cord titers and the transplacental transfer ratio were higher in the second quarter of the year. Given the inverse relationship between maternal IgG titer and transfer ratio, we propose that this period may represent a time of increased transmission and, therefore, exposure to *S. sonnei* in HCMC. This hypothesis was supported by the observed increase in maternal IgM titer between May and July (representing acute infection), suggesting that mothers’ existing immune response may be naturally boosted during this time. If *S. sonnei* transmission in HCMC is more common between April and June then infants born in during this time are likely better equipped against *S. sonnei* exposure at birth as the cord titers are highest during this season. However, annual trends are difficult to evaluate from our yearlong dataset.

There were several limitations with this study. Firstly, the infrequent early blood samples from infants prevented high-resolution temporal analyses regarding maternal half-life duration and survival analysis of the waning of maternal IgG. Next, the lack of a similar cohort from a non-endemic area limits our ability to fully interpret the serology data in an epidemiological context. Furthermore, a lack of disease data prohibits an analysis of the protective effect of presence of antibody as well as a more detailed analysis of anti-*S. sonnei*-O IgG and IgM response in infants after infection. However, the major strength of this study is the cohort design and relatively limited loss to follow up which enables us to generalize our conclusions to Vietnamese infants in urban HCMC. In the future, investigations into additional protective factors against *Shigella*, such as breastfeeding, may be warranted [51].

In summary, *S. sonnei* exposure is common in HCMC and maternal IgG is readily transferred across the placenta, waning by 5 months of age in the majority of infants. In the event of licensure of a sufficiently safe and immunogenic *S. sonnei* vaccine, it would be prudent to vaccinate after the waning of maternal IgG in settings such as HCMC. Furthermore, we found that neonates have a higher concentration of IgG compared to mothers in most cases, and the ratio of transplacental transfer is inversely related to the maternal anti-*S. sonnei*-O IgG titer. Finally, we identified those likely to be more at risk of *S. sonnei* exposure in infancy to include preterm neonates and those born to mothers with lower IgG titers. Therefore, appropriate monitoring and prevention strategies can be targeted to such groups.

## Authors’ contributions

CNT, KLA, LTQN, CS and SB designed and set up the cohort study. LTPT performed the ELISAs. CT carried out the analysis. NTH, LLV, NVVC, VTD, NNMC, TTHC, HHT, TVTN, PVM and TDHN were involved in the laboratory and clinical management of the cohort study. AS, LBM, AP and CG provided *S. sonnei* antigen and protocols for the ELISA. CT, GT and SB wrote the manuscript.

## Figures and Tables

**Table 1:** Baseline characteristics of 503 Vietnamese infants enrolled in the birth cohort in 2013.

Characteristic	*n* (%), Median (IQR)
Male sex	260 (51.7)
Gestational age (weeks)	39 (38–40)
Preterm (<37 weeks)	21 (4.2)
Birth weight (kg)	3.15 (2.9–3.4)
Low birth weight (<2.5 kg)	23 (4.6)
Vaginal delivery	288 (57.3)
Breastfed during month 1	
Exclusively	215 (43.0)
Plus formula	243 (48.6)
No, only formula	42 (8.4)
Gravidity	2 (1–3)
Maternal education	
Lower secondary or below	255 (50.7)
Higher secondary or above	248 (49.3)
Maternal age (years)	28 (25–31)

**Table 2:** Demographic and socioeconomic characteristics of infants with plasma samples available from four follow up visits (0, 4, 9 and 12 months of age) and those who attended less than four visits, *n* (%).

Characteristic	<4 visits	4 visits	*p*^a^
	*n* = 211	*n* = 292	
Vaginal birth	111 (52.6)	177 (60.6)	0.073
Male infant	113 (53.6)	147 (50.3)	0.477
Infant low birthweight	9 (4.3)	14 (4.8)	0.779
Any previous children	128 (60.7)	185 (63.4)	0.539
Maternal age ≥28 years	109 (51.7)	150 (51.4)	0.949
Low maternal education	106 (50.2)	142 (48.6)	0.722
Household crowding	116 (55)	185 (63.4)	0.059
Infant cord log_10_ titer > 2.3^b^	122 (57.8)	151 (51.7)	0.175
Preterm (<37 weeks)	9 (4.3)	12 (4.1)	1.00
Breastfed during month 1			
Exclusively	89 (42.6)	126 (43.3)	0.726
Plus formula	100 (47.8)	143 (49.1)	
Formula + food	20 (9.6)	22 (7.6)	
Mother ethnic minority	21 (10)	21 (7.2)	0.269
Father ethnic minority	26 (12.3)	24 (8.2)	0.129
Watersource			
Piped home	144 (68.2)	208 (71.2)	0.73
Bottled	63 (29.9)	80 (27.4)	
Other	4 (1.9)	4 (1.4)	

a *p*-value derived from chi-square or Fisher's exact test.

b Median.

**Table 3:** Geometric mean titers (GMT) of anti-*S. sonnei*-O IgG in maternal and cord plasma and the ratio of cord:maternal IgG titer.

Category	*n* Pairs	Maternal IgG	Cord IgG	Median ratio	Comparison^a^	
		GMT (range)	GMT (range)	(range)	*p* value	Group
Total	503	167.4 (3.75–2553.7)	230.8 (0.22–3687.6)	1.33 (0–12.4)		
Gestational age						
<37 weeks (1)	21	190.8 (48.5–545.8)	197.4 (49.5–546.8)	1.13 (0.4–2.6)	0.019	1:2
37–40 weeks (2)	549	166.5 (3.7–2553.7)	232.1 (0.22–3687.6)	1.35 (0–12.4)	0.130	1:3
>40 weeks (3)	23	165.7 (22.3–1175.0)	237.7 (68.8–1163.3)	1.33 (0.6–6.2)	1.000	2:3
Sex^b^						
Female	243	149.2 (8.2–2553.7)	209.9 (0.22–2140.9)	1.37 (0–12.4)	0.177	
Male	260	186.4 (3.7–2524.2)	252.1 (23.1–3687.6)	1.28 (0.3–11.2)		
Birthweight						
<2500 g	23	150.8 (26.1–1280.1)	195.9 (42.4–2140.9)	1.25 (0.5–3.8)	0.515	
≥2500 g	480	168.3 (3.7–2553.7)	232.6 (0.22–3687.8)	1.33 (0–12.4)		
Gravidity						
1	190	150.5 (3.7–2553.7)	216.7 (0.2–2824.8)	1.39 (0–12.4)	0.066	
>1	313	178.6 (15.7–2524.2)	239.7 (23.1–3687.6)	1.3 (0.3–11.2)		
Maternal age^b^, ^c^						
<28 years	244	138.7 (3.7–1530.9)	204.5 (1.1–2824.8)	1.36 (0.3–12.4)	0.065	
≥28 years	259	199.9 (20.6–2553.7)	258.6 (0.22–3687.6)	1.29 (0–8.3)		
Maternal education						
Lower secondary or below	255	179.9 (3.7–2553.7)	250 (26.7–3687.6)	1.29 (0.3–12.4)	0.449	
Higher secondary or above	248	155.5 (15.7–2524.2)	212.5 (0.22–3206.5)	1.35 (0–11.2)		
Maternal IgM^c^						
≤1.37	253	158 (3.7–2553.7)	268.4 (22.1–2824.8)	1.66 (0.37–12.4)	<0.001	
>1.37	250	177.5 (15.7–2524.2)	203.8 (21.6–3687.6)	1.16 (0.3–11.2)		
Season^c^						
January–March (1)	89	148.2 (3.7–2553.7)	244.4 (34.1–2824.8)	1.7 (0.6–11.8)	0.024	1:2
April–June (2)	133	174.3 (8.2–1596.0)	358.3 (65.3–3687.6)	1.9 (0.4–12.4)	<0.001	1:3
July–September (3)	161	162.6 (15.7–2524.2)	181.8 (22.1–3206.5)	1.14 (0.3–11.2)	<0.001	1:4
October–December (4)	120	182.3 (33.5–1969.7)	187 (0.22–2123.2)	1.12 (0–5.8)	<0.001	2:3
					<0.001	2:4
					1.000	3:4

a *p*-values comparing ratio of transfer between categories of each characteristic, *p*-values corrected for multiple comparisons are shown with groups indicated in parentheses next to the group name.

b Significant difference in log_10_ titers of maternal plasma per category.

c Significant difference between log_10_ titers of cord samples per category; titers are shown in ELISA Units (EU).

**Table 4:** Univariate and multiple linear regression measuring the effect of different covariates on the outcome of log_10_ cord anti-*S. sonnei*-O IgG titer.

Characteristic	Cord blood IgG titer			
	Univariate		Adjusted	
	Beta	*p*	Beta	*p*
Cord:maternal IgG ratio	0.595	1.36	<0.001	
Infant				
Male sex	0.07	0.067	0.00	0.850
Gestational age	0.04	0.006	0.01	0.549
Birthweight	0.08	0.102	−0.01	0.504
Maternal				
Age	0.01	0.001	0.00	0.246
Low education	−0.06	0.123	0.01	0.410
Gravidity	0.03	0.088	0.00	0.921
Log_10_ IgM	−0.14	0.006	−0.01	0.798
Log_10_ IgG	0.76	<0.001	1.01	<0.001
Season				
January–March	0.13	0.013	0.02	0.170
April–June	0.29	<0.001	0.08	<0.001
July–September	1.00	–	1.00	–
October–December	0.04	0.417	0.01	0.637

Beta values represent the slope of the linear association and *p*-values demonstrate whether the slope is significantly different from the null hypothesis of 0.
